# Restoration of diaphragmatic function after diaphragm reinnervation by inferior laryngeal nerve; experimental study in rabbits

**DOI:** 10.1186/1465-9921-7-17

**Published:** 2006-01-27

**Authors:** Stephane Derrey, Eric verin, Annie Laquerrière, Angelique Boishardy de Barros, Yann Lacoume, Pierre Fréger, Jean Paul Marie

**Affiliations:** 1 Experimental Surgery Laboratory, Rouen University, School of Medicine, France; 2 Department of Neurosurgery, Rouen University Hospital, Charles Nicolle, France; 3 GRHV-EA 3830. Groupe de recherche sur le handicap ventilatoire (Ventilatory insufficiency research group), IFRMP 23, Rouen University, France; 4 Department of Physiology, Rouen University Hospital, Charles Nicolle, France; 5 Department of Pathology, Rouen University Hospital, Charles Nicolle, France; 6 Department of Otolaryngology Head and Neck Surgery, Rouen University Hospital, Charles Nicolle, France

## Abstract

**Objectives:**

To assess the possibilities of reinnervation in a paralyzed hemidiaphragm via an anastomosis between phrenic nerve and inferior laryngeal nerve in rabbits. Reinnervation of a paralyzed diaphragm could be an alternative to treat patients with ventilatory insufficiency due to upper cervical spine injuries.

**Material and method:**

Rabbits were divided into five groups of seven rabbits each. Groups I and II were respectively the healthy and the denervated control groups. The 3 other groups were all reinnervated using three different surgical procedures. In groups III and IV, phrenic nerve was respectively anastomosed with the abductor branch of the inferior laryngeal nerve and with the trunk of the inferior laryngeal nerve. In group V, the fifth and fourth cervical roots were respectively anastomosed with the abductor branch of the inferior laryngeal nerve and with the nerve of the sternothyroid muscle (originating from the hypoglossal nerve). Animals were evaluated 4 months later using electromyography, transdiaphragmatic pressure measurements, sonomicrometry and histological examination.

**Results:**

A poor inspiratory activity was found in quiet breathing in the reinnervated groups, with an increasing pattern of activity during effort. In the reinnervated groups, transdiaphragmatic pressure measurements and sonomicrometry were higher in group III with no significant differencewith groups IV and V.

**Conclusion:**

Inspiratory contractility of an hemidiaphragm could be restored with immediate anastomosis after phrenic nerve section between phrenic nerve and inferior laryngeal nerve.

## Background

Approximately 20% of patients with acute cervical spinal cord injuries will require some form of mechanical ventilatory support due to bilateral diaphragmatic paralysis. For these patients, an alternative technique could be diaphragm reinnervation [1-5]. Among the nerves tested to perform diaphragm reinnervation, the inferior laryngeal nerve, was certainly the optimal donor nerve because of its similarities with the phrenic nerve [4,5]. Guth et al., [4] showed poor results with this technical approach in rats and monkeys but in cats, Baldissera et al. found encouraging one [5]. These studies demonstrated that diaphragmatic reinnervation was technically possible, but never evaluated the reinnervated diaphragmatic function. Nevertheless, even if the inferior laryngeal nerve contains a majority of inspiratory axons in its abductor branch (which innervates the posterior cricoarythenoid muscle) [6], it also contains a majority of expiratory axons in its adductor branch, which could impair inspiratory diaphragm contraction [5]. Theoretically, the utilization of pure inspiratory nerves should improve the quality of the diaphragm reinnervation. The abductor branch of the inferior laryngeal nerve as a donor nerve should theoretical be the best option. Another option for diaphragm reinnervation could be the sterno-thyroid branch of the hypoglossal nerve, which also contains a majority of inspiratory axons [7,8].

The aim of the study was then, to perform in rabbits, an unilateral diaphragmatic reinnervation of a right hemidiaphragm paralyzed by a section of the right phrenic nerve in the neck and to evaluate the restoration of diaphragmatic function after reinnervation. Three different donor nerves were used: 1- the inferior laryngeal nerve, 2- the abductor branch of the inferior laryngeal nerve, 3- the sterno thyroid branch of the hypoglossal nerve coupled with the abductor branch of the inferior laryngeal nerve.

## Materials and methods

### Animals and groups

**Figure 1:**
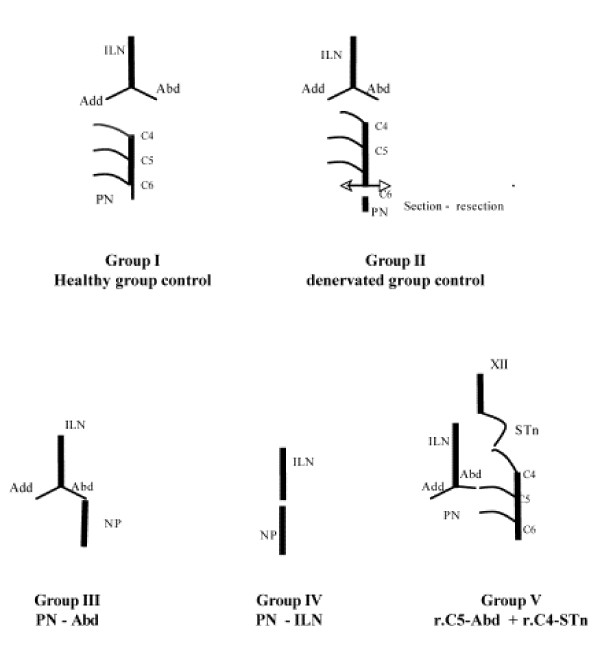
Schematic surgery representation of the different groups. ILN: inferior laryngeal nerve; Add/Abd: adductor and abductor branches of inferior laryngeal nerve; PN: phrenic nerve; r.C4-r.C5-r.C6: C4-C5 and C6 cervical roots of the phrenic nerve; XII: hypoglossal nerve; ST:, nerve of sternothyroid muscle originated from the cervical roots of the hypoglossal nerve.

A total of 35 New Zealand rabbits (C.E.G.A.V., Saint Mars d'Egrenne, France) were included in this study. The animals were divided in five groups of seven rabbits each (fig. 1). Two groups (GI and GII) were used as controls and three (GIII, GIV and GV) were designed as study groups with three different reinnervation protocols.

-GI: rabbits were left intact and used as controls (healthy group control).

-GII: a section and resection of the entire right phrenic nerve (PN) in the neck was performed (denervated group control).

-GIII: the right PN trunk was sectioned in the neck below the 6^th ^cervical root and was immediately anastomosed to the abductor branch of the right inferior laryngeal nerve (ILN).

-GIV: the right PN trunk was sectioned in the neck below the 6^th ^cervical root and was immediately anastomosed to the right ILN dissected inside the larynx.

-GV: the right 4^th^, 5^th ^and 6^th ^(r.C4, r.C5, r.C6) cervical roots which constitute the right PN were sectioned in the neck. The r.C4 was anastomosed to the abductor branch of the ILN and r.C5 (r.C5 – r.C6 anastomosis) was anastomosed to the nerve of the sternothyroid (STn) (branch of the hypoglossal nerve) muscle.

In this study, the five groups were classified as: GI (healthy group), GII (denervated group), GIII (PN – Abd), GIV (PN – ILN) and GV (r.C5-Abd + r.C4-STn).

The experiments were carried out in accordance with the French code of conduct concerning laboratory animals *(university license: A76-450-05, surgeon license: 76.A.21, and Ethical committee for Animal Experimentation in Normandy-France N/01-11-03-04)*.

### Surgical procedure

The rabbits were premedicated with Valium^® ^(10 mg/kg, IM) and anesthetized with ketamine hydrochloride (12.5 mg/kg) and chlorpromazine hydrochloride (0.625 mg/kg). Depth of anesthesia was adjusted to maintain abolition of the corneal reflex and retain spontaneous breathing.

A medial cervicotomy was performed under aseptic conditions. The right PN and the cervical roots were exposed under high magnification (Zeiss, Germany). Identification of the PN was controlled by abdominal expansion induced by electrical stimulation of the PN (2 mA) (Vari-Stim RIII, Medtronic Xomed, Jacksonville, FL). In rabbit, PN issues from r.C4, r.C5 and r.C6. An accessory PN often originates from r.C6. PN was sectioned in the neck below r.C6 [9]. The right ILN was located in the tracheo-esophageal groove and followed down to the posterior cricoarytenoid muscle. The larynx was exposed and rotated along its longitudinal axis to expose the terminal branches of the right ILN. The adductor branch was sectioned and ligated to prevent any reinnervation with this branch. Prior to sectioning abductor branch, its functional identity was verified via electrical stimulation. ILN and its branches were then passed under the sternocleidomastoid muscle. In the third group (GIII), the PN was anastomosed to the abductor branch. In the fourth group (GIV), a PN to ILN anastomosis was performed. The surgical procedure in the fifth group was in fact different. After identification of the r.C4, r.C5 and r.C6, r. C5 was anastomosed to the abductor branch of ILN and r.C4 was anastomosed to the nerve of the sternothyroid muscle. The nerve of the sternothyroid muscle issues from the descending branch of the hypoglossal nerve, innervates the sternothyroid muscle and has an accessory inspiratory activity. End-to-end perineural sutures were performed with 10.0 nylon thread (Ethylon™). After cleaning with Betadine™, the wound was closed without drainage. Animals received postoperative antalgic treatment (paracetamol) for three days after surgery. From surgery to evaluation, animals were provided with water and food ad libitum.

### Evaluation

The operated animals were evaluated between the fourth and fifth month after surgery. Thereafter, animals were conditioned under general anesthesia (ketamine hydrochloride and chlorpromazine hydrochloride), and maintained under slight anesthesia to collect the data.

#### Animal conditioning for evaluation

The rabbits were restrained supine, on a heated table. Under general anesthesia, via a medial cervicotomy, a tracheostomy was performed and the trachea was cannulated with a 4 mm ID endotracheal tube. A #00 Fleisch pneumotachograph (Lausanne, Switzerland) was connected to the endotracheal tube and to a transducer (Statham PM 197, range ± 0.01 PSI; Oxnard, CA). A balloon catheter 50 mm long (Atlan, 4.0 mm external diameter, 2.6 mm internal diameter (Marquat Genie Biomedical, Boissy Saint Leger, France) was introduced transorally into the inferior part of the thoracic esophagus and was connected to a pressure transducer (Statham PM 6, range ± 2.5 PSI) [10]. Midline laparotomy was then realized. The two external jugular veins were isolated in the neck. Two cardiac stimulation electrodes (1.5 mm diameter; four electrodes 2.0 mm high, 10.0 mm between each electrode, ref 002943, Bard, Trappes, France) were introduced into both external jugular veins [10]. The electrodes were advanced 2 – 4 cm into the upper chest in order to stimulate each phrenic nerve without foreleg contraction. Electrical stimulation was 0.5 s long train of rectangular pulses with pulse duration of 0.2 ms at 100 Hz. Intensity used was always superior (1.25 time) to the supra maximal intensity based on Pes amplitude and was used for all subsequent stimulations. Pairs of home made hocked wire (10 mm apart) electrodes were inserted into the sternal, midcostal and posterior costal regions of both hemidiaphragms. EMG signals were band-pass filtered (2–20 kHz) and amplified with a recorder (Viking, Nicolet, Madison, WI). An integrator was connected (Gould, Instrument System, Valley View, OH) in order to quantify the EMG signals. Length changes of the diaphragm muscle segments were measured during breathing using sonomicrometry. Sonomicrometer (sonomicrometer 120, Triton Technology Inc, San Diego, CA) measured the distance between pairs of small transducers implanted in muscles or similar tissues. The distance was determined by measuring the transit time of ultrasound between the pair of transducers. The time was converted to an equivalent distance. Via the laparotomy, pairs of piezoelectric crystals (Bioseb, segment length small 1.0 mm, 5 MHz, Chaville, France) were positioned 5 and 15 mm apart in sternal, mid-costal and posterior costal regions of both hemidiaphragm. The crystals were carefully aligned along the longitudinal axis of muscle fibers and held in place with purse-string sutures.

All parameters were displayed on an Apple computer using an acquisition card (MacLab/8e) and Chart V.3.4.4 software.

#### Data acquisition

##### Esophageal pressure

Because the abdomen was opened, esophageal pressure was equal to trans diaphragmatic pressure [11]. After animal conditioning, a slight anesthesia with antalgic medication (paracetamol) was maintained. Pes was measured during quiet breathing (5 minutes at least after ketamine hydrochloride re-injection), during prolonged tracheal occlusion against occluded tracheal cannula and during tetanic phrenic nerve stimulation. When Pes was measured during maximal inspiratory effort, Pes was considered PImax. Occlusion began at end of expiration (indicated by airflow visualisation), assimilated to functional residual capacity and stopped after three maximal consecutive inspiratory efforts. After recovery of quiet breathing, electrical stimulations of phrenic nerves were performed on the right side, on the left side and simultaneously on both sides. All stimulations were supramaximal and performed with the tracheal cannula occluded. Highest value was retained after three reproducible and consecutive values.

##### Electromyogram (EMG)

Paired of home made hocked wire electrodes were successively inserted in sternal, mid costal and posterior costal on both hemi diaphragms. Activity was recorded during quiet breathing 5 minutes after the last stimulation and during prolonged tracheal occlusion. To analyze EMGs, a qualitative score was determined from 0 to 3 using the following scale [12]; 0: unrythmed tracing, without increase during inspiration, 1: rythmed tracing with inspiratory increasing, but poor tracing (neurogen), 2: rythmed tracing with richer activity, 3: rythmed tracing, very rich, constituting an interference pattern, similar to a maximal intentional activity. A quantitative scale was also used after EMG integration to quantify EMG signals [13]. As regards the integrated EMG, the highest value obtained at the peak of the integrated curve was retained. Means of qualitative and quantitative scores were calculated in each group during quiet breathing and prolonged tracheal occlusion. In each group, the global electrical activity of each hemidiaphragm was determined by the mean of the qualitative scores obtained in the three regions. When an expiratory activity was recorded, only the qualitative scale was used [12].

##### Sonomicrometry

Crystals of sonomicrometry were inserted in two regions (sternal and midcostal) on both sides. Changes in fiber length were recorded during quiet breathing, prolonged tracheal occlusion, and supramaximal phrenic nerve stimulations (uni and bilateral). The highest value among three consecutive measurements was retained. Sonomicrometric results were expressed as a percentage of stretching or shortening compared with the reference length, measured at the end of the expiration. EMG activity, esophageal pressure and sonomicrometric measurements were recorded successively always in the same order.

### Methodological verifications

Following the above mentioned explorations, prior to dissection of the cervical region, abdominal expansion was verified during a stimulation of the vagus nerve, from which the ILN is issued. After this methodological verification, the cervical region was dissected in order to control stimulation of the nerve above the anastomosis. Animals were excluded from the statistical analysis when the stimulation of a nerve different of inferior laryngeal nerve or its branch or sternothyroid branch of the hypoglossal nerve, provided a contraction of the right hemidiaphragm.

### Histology

Lastly, animals were sacrificed with pentobarbital overdose by an intravenous injection. Longitudinal sections were performed in the sternal, mild costal and posterior costal regions of the right hemidiaphragm. Sections were immediately placed into a 10% formalin buffer solution, then embedded in paraffin. The micro sections were stained with Haematoxylin-eosin. Signs of denervation of the right hemidiaphragm were recorded in each group using the following parameters: fiber size, fiber shape (angulated or rounded fibers), nuclear internalisations, fiber atrophy and necrotic fibers. A denervation score was determined in each region of right hemidiaphragm. The lesions were finally classified as: none (0), slight (1), moderate (2) and severe (3). Mean values were calculated for each animal and each group.

### Statistical analysis

Statistical analysis of the functional parameters (EMG, esophageal pressure and sonomicrometry measurements) was performed using NCSS software (Number Cruncher Statistical Systems, Dr JL. Hintze, Kaysville, UT). Non-parametric tests (Kruskal – Wallis, one way ANOVA) were performed to compare EMG scores, trans diaphragmatic pressure and histological scores. For all parameters, *p *values were considered as significant if < 0.05. When *p *< 0.05, each group was compared with the four other groups using routine tests (*p *< 0.05).

## Results

After surgery recovery, no rabbit had respiratory distress or died. Three rabbits developed a well tolerated cervical abscess on an epidermoid cyst.

Thirty-five rabbits were operated on and 26 were analyzed. Four animals died just prior to evaluation and two during premedication just before the evaluation. Five animals were excluded from the statistical analysis because of absence of any sign of reinnervation in the right hemidiaphragm for reinnervated group and three because the reinnervation was not completed by the surgical technique, demonstrated by right absence of diaphragmatic response after stimulation of the vagus nerve at the time of evaluation. In two animals, this residual innervation was due to a branch originating from the sixth cervical root discovered after cervical dissection. However, the origin of the innervation in one was not established. For statistical analysis, only surgical success was taken into consideration (i.e. when the reinnervation was supplied by the surgical technique) in order to specifically compare the quality of the reinnervation induced by each transposed nerve. Thus, 26 rabbits were included in the statistical analysis: 7 in GI (healthy group), 6 in GII (denervated group), 4 in GIII (PN – Abd), 5 in GIV (PN – ILN) and 4 in GV (r.C5-Abd + r.C4-STn).

### Restoration of diaphragmatic innervation

#### Electromyogram

##### Global electrical activity (fig. 2)

**Figure 2:**
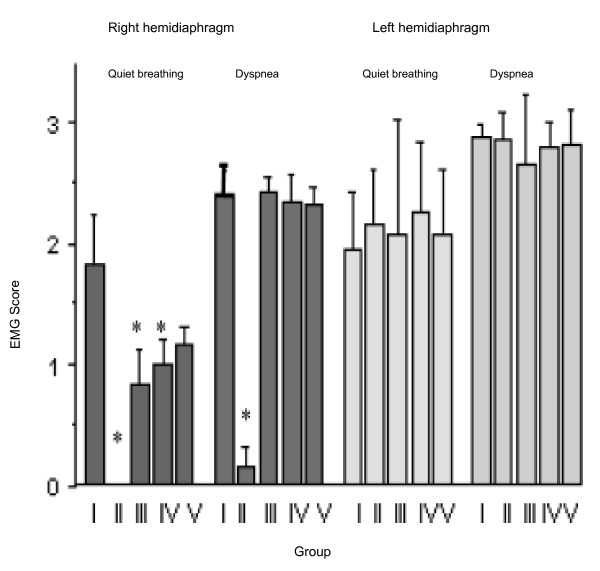
Global electrical activity (EMG score) obtained in the different group, during quiet breathing and tracheal occlusion for the right and left hemidiaphragm. In right hemidiaphragm of group II, no electrical activity was observed during quiet breathing or a very poor activity.

During quiet breathing, the means of EMG scores (qualitative scale) were similar in the three reinnervated groups, but lower than in group I (p < 0.05) and higher than in group II (p < 0.05). During tracheal occlusion, it was not different between GI and the three reinnervated groups and higher than GII (p < 0.05). One rabbit in group II (GII#1) showed residual inspiration activity during tracheal occlusion in the mid and posterior costal regions of the right hemidiaphragm.

##### Regional EMG activity (sternal, mid-costal and posterior costal region)(fig. 3)

**Figure 3:**
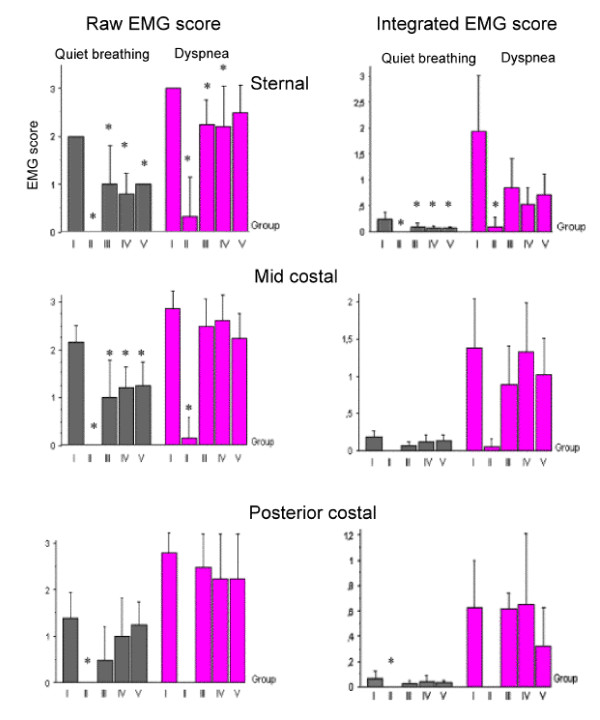
Right diaphragmatic electromyographic score (left, calculated after analysis of raw data; right, calculated after analysis of integrated EMG) in each regions of the right hemidiaphragm (right sternal region, right mild-costal region, right posterior costal region); those different EMGs were obtained during quiet breathing or during dyspnee (occlusion). * significant difference with the group I (Z>1,96 with p < 0,05).

**Figure 4:**
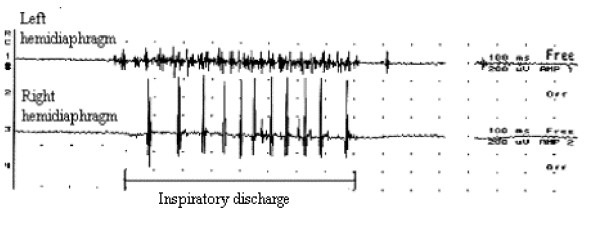
Costal (left and right) inspiratory activity recorded during quiet breathing in one animal of group IV, 4 months postoperatively. In the right hemidiaphragm, the activity was poor and neurogen (EMG score = 1). In the left hemidiaphragm, the inspiratory activity was normal (EMG score = 2).

During quiet breathing, inspiratory activity was poorer, as illustrated in representative example in figure 4, in the reinnervated groups than in group I (healthy group). With the qualitative scale, the differences were significant in the sternal and mid costal regions. In posterior costal region, higher values were measured in group I (healthy group). With the quantitative scale, the only significant difference between group I and the three reinnervated groups was found in the sternal region of the right hemidiaphragm.

During tracheal occlusion, with the qualitative scale, difference between group I (control group) and the groups II (denervated group), III (PN – Abd) and IV (PN – ILN) were considered significant. In mid costal and posterior regions, during tracheal occlusion, inspiratory activity increased in each reinnervated groups without any significant difference with the group I. No statistical difference was found between the three reinnervated groups during tracheal occlusion, but all reinnervated groups showed a significant difference from group II (denervated controls).

#### Right hemi diaphragm histology

The mean denervation score was equal to zero in the healthy group. The highest score of denervation was in group II (denervated group). Among the six denervated animals in group II, three had a severe denervation, whereas three had a moderate denervation. In the three reinnervated groups, no statistical differences were found between the three reinnervated groups and GI, the denervation scores were lower than in group II (denervated group). In groups III (PN – Abd) and IV (PN – ILN), the denervation scores were higher in the right sternal region.

### Restoration of diaphragmatic function

#### Transdiaphragmatic pressure

Means and standard deviations are displayed in the Table I. During quiet breathing, no statistical difference was found between the groups. During right supramaximal stimulation, Pes was near to nul in group II (denervated group). In GIV and GV, Pes during right phrenic nerve stimulation were lower than in group I (p < 0.05) and not different between GI and GIII. During left stimulation, the lowest values were observed in the groups I (healthy group), III (PN – Abd) and V (r.C5-Abd + r.C4-STn). In contrast, Pes was higher in the group IV and in group II compared to GI (p < 0.05). During bilateral supramaximal stimulation, Pes was lower in GII and GV compared to GI (p < 0.05) and not different between GI, GIII and GIV.

**Table 1:** Transdiaphragmatic pressures.

	Rest	PI max	R PNS	L PNS	B PNS
	cmH2O	cmH2O	cmH2O	cmH2O	cmH2O
G I	1.7 ± 0.6	21.5 ± 2.8	10.5 ± 1.8	9.3 ± 0.8	15.7 ± 3.4
G II	2.6 ± 1.1	20.5 ± 3.1	na	12.4 ± 1.2*	12.4 ± 1.2
G III	1.7 ± 0.3	27.4 ± 4.5*	6.1 ± 1.2*	10.6 ± 2.2	16.3 ± 2.3
G IV	2.3 ± 0.8	27.0 ± 1.0*	8.7 ± 4.0	15.8 ± 4.8*	16.9 ± 4.7
G V	1.9 ± 0.8	20.0 ± 4.7	6.4 ± 2.0*	10.6 ± 2.0	11.8 ± 2.2*

Values are means ± SD and are expressed in cm H_2_O. Rest: Esophageal pressure (Pes) during quiet breathing; PImax: Pes during prolonged occlusion tracheostomy cannula; R PNS: Pes obtained by supramaximal stimulation of the right phrenic nerve; L PNS: Pes obtained by supramaximal stimulation of the left phrenic nerve; B PNS: Pes obtained by bilateral supramaximal stimulation of the phrenic nerve; na, not available; *: statistical difference with the healthy control group (GI) (Z > 1.96 or *p *< 0.05).

-GI: healthy group control; -GII: section and resection of the entire right phrenic nerve (PN) in the neck (denervated group control); -GIII: the right PN trunk sectioned in the neck below the 6^th ^cervical root and immediately anastomosed to the abductor branch of the right inferior laryngeal nerve (ILN); -GIV: right PN trunk sectioned in the neck below the 6^th ^cervical root and immediately anastomosed to the right ILN; -GV: the right 4^th^, 5^th ^and 6^th ^(r.C4, r.C5, r.C6) cervical roots sectioned in the neck, r.C4 anastomosed to the abductor branch of the ILN and r.C5 anastomosed to the nerve of the sternothyroid muscle.

#### Sonomicrometry

**Figure 5:**
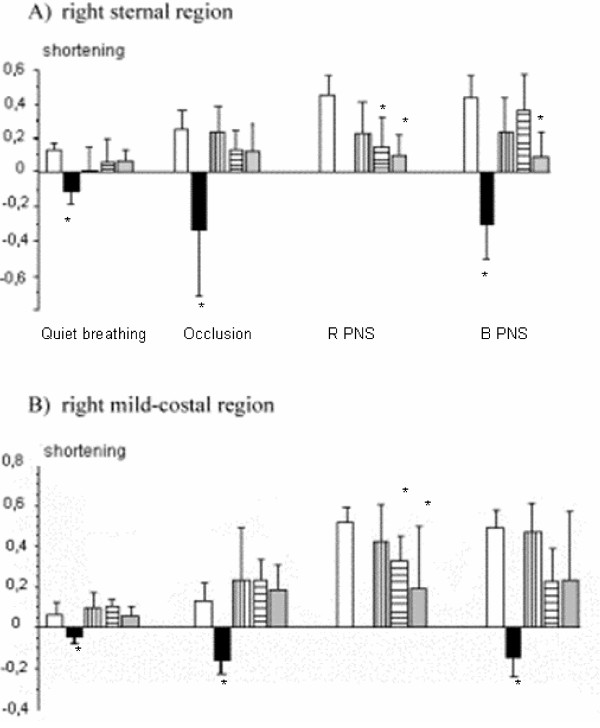
Sonomicrometric measurements in the sternal region (A) and in the mid-costal region (B) of the right hemidiaphragm in each group. Occlusion: measurement performed during prolonged tracheal occlusion; R PNS: measurement performed during a right supramaximal stimulation of the right phrenic nerve; B PNS: measurement performed during a bilateral supramaximal stimulation of the phrenic nerve; % shortening, percentage of shortening in relation to the resting length of the muscular fiber measured at the end of the expiration.

In the three reinnervated groups, sonomicrometric values were positive (i.e diaphragmatic muscular fiber shortening) during quiet breathing, prolonged tracheal occlusion or phrenic nerve stimulations and were negative (i.e. diaphragmatic muscular fiber lengthening) in denervated group (GII) except in one animal (Fig. 5). During quiet breathing and tracheal occlusion, muscular fiber shortenings in the three reinnervated groups were lower than in group I (healthy group) but without any significant difference. No difference was observed between reinnervated groups. During right phrenic nerve stimulation, shortenings in groups IV and V were statistically lower than in group I (healthy group), and statistically not different between GI and GIII. During bilateral phrenic nerve stimulation, right fiber shortenings in the mid-costal region were lower in groups IV (but did not reach statistical difference) and V and values of groups I and III were relatively equal. Only one rabbit in the group II showed shortening during prolonged tracheal occlusion. The same animal showed EMG activity during tracheal occlusion. However, in this animal, right phrenic nerve stimulation provided no right hemidiaphragm contraction.

### Paradoxical expiratory activity

**Figure 6:**
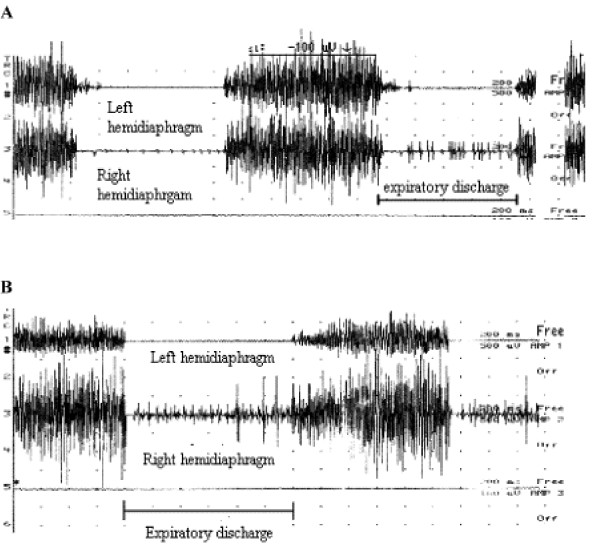
(A), Expiratory activity recorded during prolonged tracheal occlusion in the costal region of the right hemidiaphragm in one animal of group V (expiratory EMG score = 1). No expiratory activity was found in the left hemidiaphragm. (B), Expiratory activity recorded during prolonged tracheal occlusion in the costal region of the right hemidiaphragm in one animal of group IV (expiratory EMG score = 2). No expiratory activity was found in the left hemidiaphragm.

In each reinnervated group, several animals showed an expiratory electrical activity (one illustrative example is depicted in fig. 6). Two were in group III (PN – Abd), three in group IV (PN – ILN) and one in group V (r.C5-Abd + r.C4-STn). This activity was not recorded in group I and in the left hemidiaphragm of the three reinnervated groups. In group III (PN – Abd), two had a poor expiratory activity which was only recorded during prolonged tracheal occlusion (score = 1 with the qualitative scale). This activity was found in the right sternal region or in the right sternal and mid-costal regions. In the group IV (PN – ILN), 3 animals showed an expiratory activity. In one animal, this activity was poor and only recorded in the right sternal region during tracheal occlusion, and in the other this activity was higher (score = 2). Lastly, one rabbit in the group V had an expiratory activity (score = 1) recorded only during prolonged tracheal occlusion in the right mid-costal and posterior costal regions.

## Discussion

Our results confirmed that the inspiratory activity of a right paralyzed hemidiaphragm can be restored in rabbit by the anastomosis between the right phrenic nerve and the right inferior laryngeal nerve or its branch and demonstrated a restoration of diaphragmatic strength close to normal, even if no statistical difference was found between the three reinnervation modalities.

### Methodological considerations

#### Justification of the method

Due to its respiratory similarities with the phrenic nerve (its activity begins a few milliseconds before the phrenic nerve discharge (between 40 et 80 ms) [14] and is increase during hypercapnia and hypoxia [6]), the inferior laryngeal nerve was chosen to perform diaphragm reinnervation. The first diaphragm reinnervation was usefully done with the vagus nerve in dogs and in rats [1,3]. In 1960, Guth et al., were the first to use the inferior laryngeal nerve as a donor nerve [4] in 8 rats and 3 monkeys, with positive results in two rats and one monkey. In 1993, Baldissera et al. [5] used the inferior laryngeal nerve or its branches as donor nerves in 10 cats, but analysis of their results was quite difficult due to the limited number of animals used in each group. In contrast to our study, Baldissera et al. did not use the abductor branch alone to perform the reinnervation of a complete hemidiaphragm [5]. Some authors [2] used nerves without any respiratory activity i.e. the facial, the accessory and the long thoracic nerve, with poor results. Since, the stimulation of the transposed nerve induces a contraction of the reinnervated hemidiaphragm, these nerves were unable to induce spontaneous diaphragmatic rhythmic contractions.

Our aims were to study diaphragmatic effects of diaphragmatic reinnervation by the inferior laryngeal nerve or its branch and with the nerve of the sternothyroid muscle originating from the hypoglossal nerve, using EMGs, histology and transdiaphragmatic pressure, near as possible to diaphragmatic explorations in humans. Our approach was therefore indirect regarding muscle function, and especially pressure measurements, which test the overall diaphragmatic function. The muscle fibers approach, even if it could bring additional data, was therefore not retained in our study.

#### Transdiaphragmatic pressure measurements

In our study, as the abdomen was open, gastric pressure was considered to be identical to atmospheric pressure. Consequently, esophageal pressure was equal to the arithmetic inverse of transdiaphragmatic pressure and was considered as a measure of the diaphragmatic force [11,15] and was measured during airway occlusion, under isometric conditions [11]. Nevertheless, the pressure that the abdominal wall and content exerts on the diaphragm during inspiration has an impact on the thoracic mechanics and therefore on the pressure generated by the diaphragm contraction. Performing all esophageal measurements with an opened abdominal cavity may have altered the pressure generating capacity of the diaphragm and could explain the lack of significance during bilateral phrenic nerve stimulation.

Transdiaphragmatic pressure is measured during uni or bilateral phrenic nerve stimulation is not different using transvenous stimulations or direct stimulation of the nerve in the neck [10]. We chose supramaximal tetanic stimulation at 100 Hz frequency as previously performed in rabbits [10,16,17]. However, an unequal distribution of the reinnervation might have induced differences between the strength of the different diaphragmatic portions [13].

#### Sonomicrometry

Sonomicrometry provided reliable, accurate and objective values of changes of the muscular fiber length [18,19]. In the posterior costal region, measurements were considered unreliable, including measurements of the control group. Measurements were always performed at the end of the evaluation because of the high risk of pneumothorax. With sonomicrometry, the result during quiet breathing and prolonged tracheal occlusion were quite similar with the results of Zhan et al. [19].

### Significance of the findings

#### Reality of hemidiaphragm reinnervation

Our results demonstrated that hemidiaphragm reinnervation by inferior laryngeal nerve or its branch restored a diaphragm function near to normal regarding EMGs, transdiaphragmatic pressure, sonomicrometry and histology. In the three reinnervated groups, a good restitution of the electrical inspiratory activity in paralyzed hemidiaphragm was found. During quiet breathing, almost all reinnervated animals had a poor and neurogen activity in the three studied regions. However, during prolonged tracheal occlusion, the electrical activity increased just as far as in the healthy group. In the reinnervated animals, EMG activity was better improved in mid and posterior costal regions. Those results indicate that diaphragmatic respiratory drive was restored using the inferior laryngeal nerve or its branch, but incompletely during quiet breathing. It could be explained by a partial recruitment of the transferred nerve, completed during inspiratory effort [20]. As regards EMG, sonomicrometric measurements were better in the mid-costal region, during quiet breathing and tracheal occlusion. These results are in agreement with histological examination which found more severe denervation injuries in the sternal region in the groups III (PN – Abd) and IV (PN – ILN).

#### Expiratory activity of the reinnervated hemidiaphragm

Six rabbits showed expiratory activity, three from group IV (PN – ILN), two from group III and one from group V. We expected this type of activity in group IV, but in the two other groups it was less obvious. However, if the abductor branch contains a great majority of inspiratory axons, it can sometimes contain few expiratory axons for the interarytenoid muscle [21]. Expiratory activity was poorer in the group III (PN – Abd) compared with the group IV (PN – ILN) because expiratory axons are less numerous than in the adductor branch. An expiratory activity can also be produced from inspiratory axons under definite physiologic circumstances (coughing, phonation) and vocal cords stabilization in expiration [22,23].

#### Spontaneous diaphragmatic reinnervation

Among the six denervated animals, only one did not show complete denervation. In fact, during prolonged tracheal occlusion in this animal, residual inspiratory activity and fiber shortening were still present in the right sternal and mild-costal regions. Furthermore, histological examination did not reveal severe but only slight denervation. However, dissection of the cervical region did not permit to identify the origin of the innervation. Partial denervation or spontaneous reinnervation through the nervous section should not be considered since the stimulation of the right PN was ineffective. Two other hypotheses could be suggested: 1- spontaneous reinnervation by intercostal nerves, 2- cross innervation. Cross innervation of the diaphragm remains a subject of controversy. Results differ between authors and between animal species. In the rabbit, Rikard-Bell and Bystrzycka [24] did not observe any controlateral retrograde labelling in the cervical spine. Marie et al. [13] reported the same conclusion with functional tests. In contrast, in cats, spontaneous diaphragmatic reinnervation from left phrenic nerve has been reported [25,26] but not confirmed [27,28]. This phenomena has also been reported in rats and monkeys [4].

#### Consequence on the left hemidiaphragm of right hemidiaphragm denervation and reinnervation: Compensatory mechanisms?

The decrease in pressure generating capacity of the diaphragm may have been partly explained by the geometrical configuration of the diaphragm of the animals and might depend of the reinnervated hemidiaphragms associated to the healthy one (left hemidiaphragm) to generate negative esophageal pressure [29]. In groups II and IV, left phrenic nerve stimulation induced higher transdiaphragmatic pressure, and could have explained that it was higher during tracheal occlusion in group III and IV, and during bilateral phrenic nerve stimulation (not significant) (table I). Those results remain unclear, but could be interpreted, either as a compensation of the left hemidiaphragm to the decreased force of the right hemidiaphragm, or as a consequence of a difference of geometry in the remaining diaphragm [29].

Those compensatory mechanisms are also suggested by EMGs and sonomicrometry. Among the three reinnervated groups, global EMG in the left hemidiaphragm was poorer in group III (PN – Abd), suggesting that among the three reinnervated group, the compensation in group III was lower. Also, sonomicrometry during quiet breathing, during prolonged tracheal occlusion and during right stimulation showed that the contractility of the right hemidiaphragm was better in group III. In this group, shortening was higher and similarly equal with results of the healthy control group (group I).

### Clinical implication

In the present work, reinervation was performed immediately after the section of phrenic nerve. This model is far from what could happen in tetraplegic patients. Indeed, in these patients, the indication of a surgical reinnervation would be discussed several months after the injury, and diaphragmatic atrophy would occur. Nevertheless, our results demonstrated that hemidiaphragm reinnervation is possible using the inferior laryngeal nerve or its branch. Prior to the application of this technique in humans, some problems should be resolved. In order to obtain conditions closer to humans, delayed reinnervation, if possible in presence of spinal lesions, has to be evaluated. However, the choice of the inferior laryngeal nerve as a donor nerve has three disadvantages. First, a vocal cord paralysis is induced by the diversion of the inferior laryngeal nerve toward the diaphragm. In fact, the unilateral section of the inferior laryngeal nerve provides a moderate dysphonia, some transitory tracheal aspiration, without any respiratory distress. Those symptoms can be solved by endoscopic [30] or surgical medialisation [31] of the paralyzed vocal cord or laryngeal reinnervation [32]. Second, is the small number of axons in inferior laryngeal nerve compared with the phrenic nerve (in cats, inferior laryngeal nerve contains approximately 400 myelinated axons as the superior root of phrenic nerve) [33]. Lastly, the inferior laryngeal nerve contains inspiratory and expiratory axons whereas the phrenic nerve contains only inspiratory axons [6].

## Conclusion

inspiratory diaphragmatic strength of a paralyzed hemidiaphragm can be restored in rabbits by reinnervation with inferior laryngeal nerve or its abductor branch. In the future, diaphragm reinnervation could become a useful alternative procedure to reduce respiratory disability of tetraplegic patients. Nevertheless, this technique should now be tested on cervical spinal cord injury model in animals before being proposed in human.

## Disclosure

This work was supported by research grants from the "Fondation de l'Avenir", 28 rue Beaumier, 75014 Paris, France (ET2-114).
